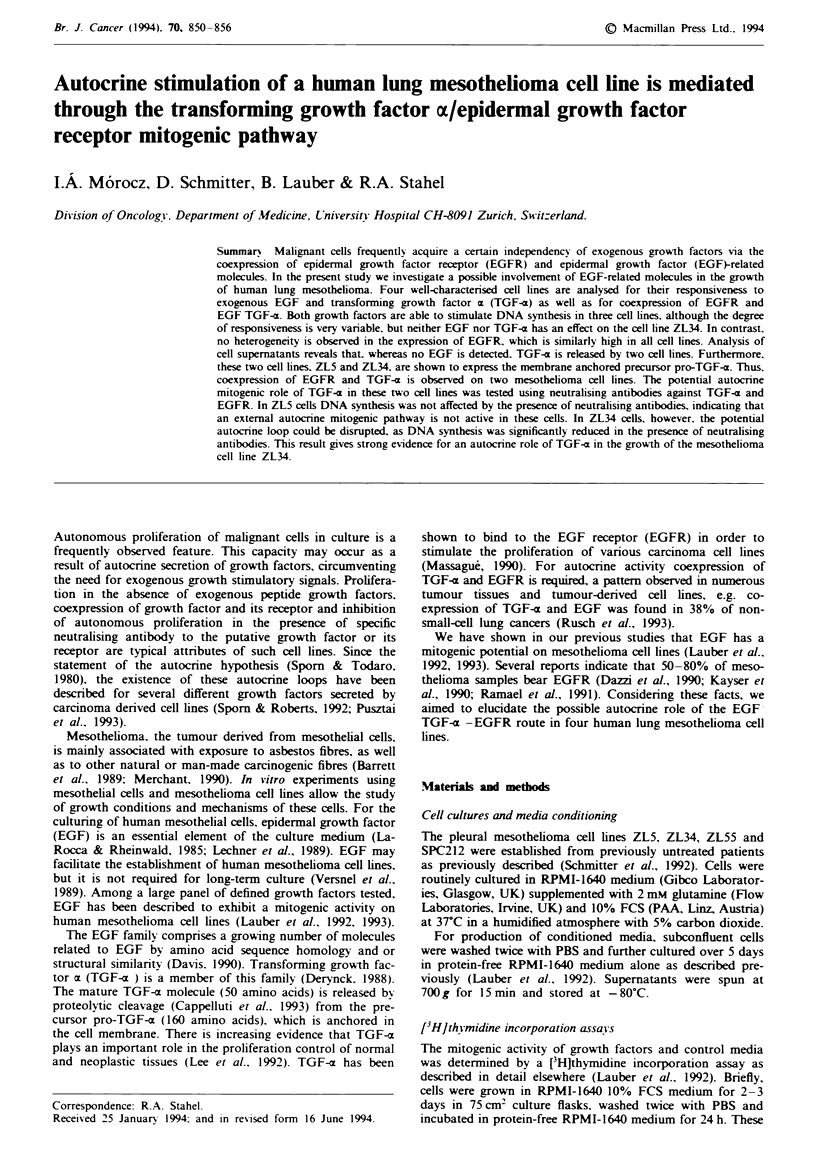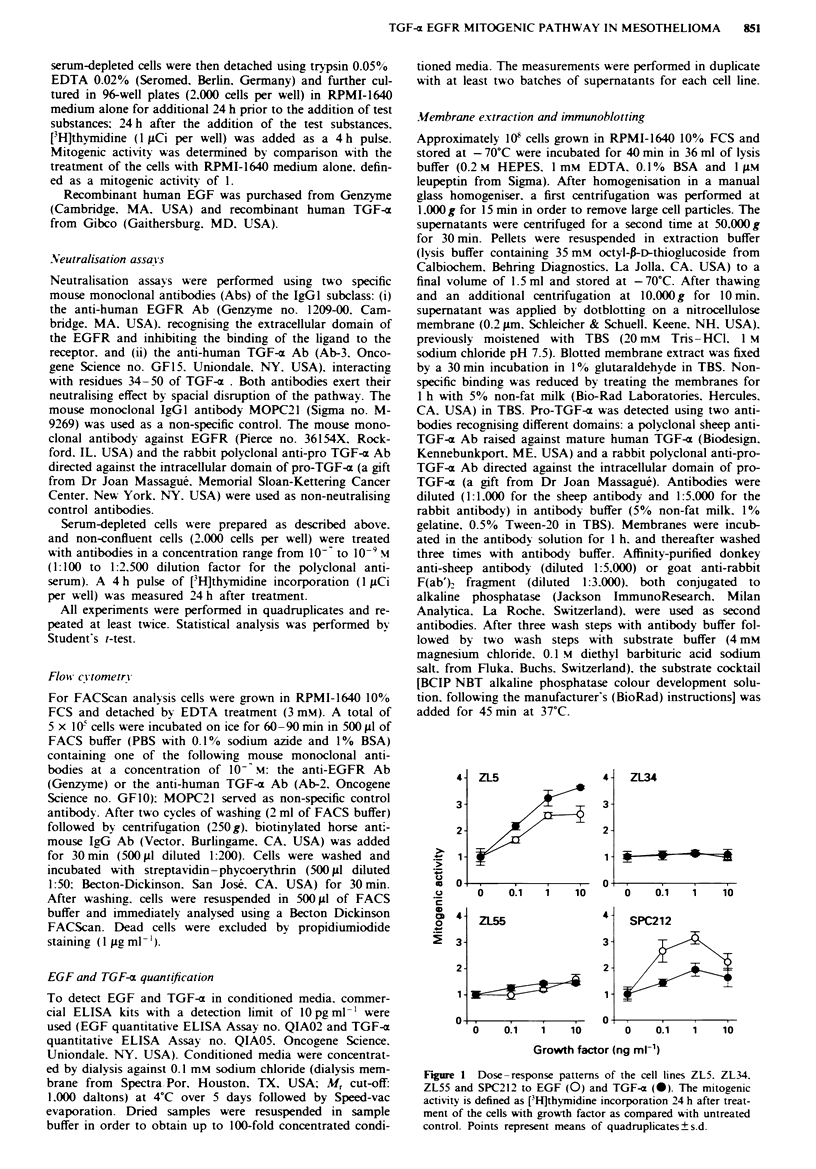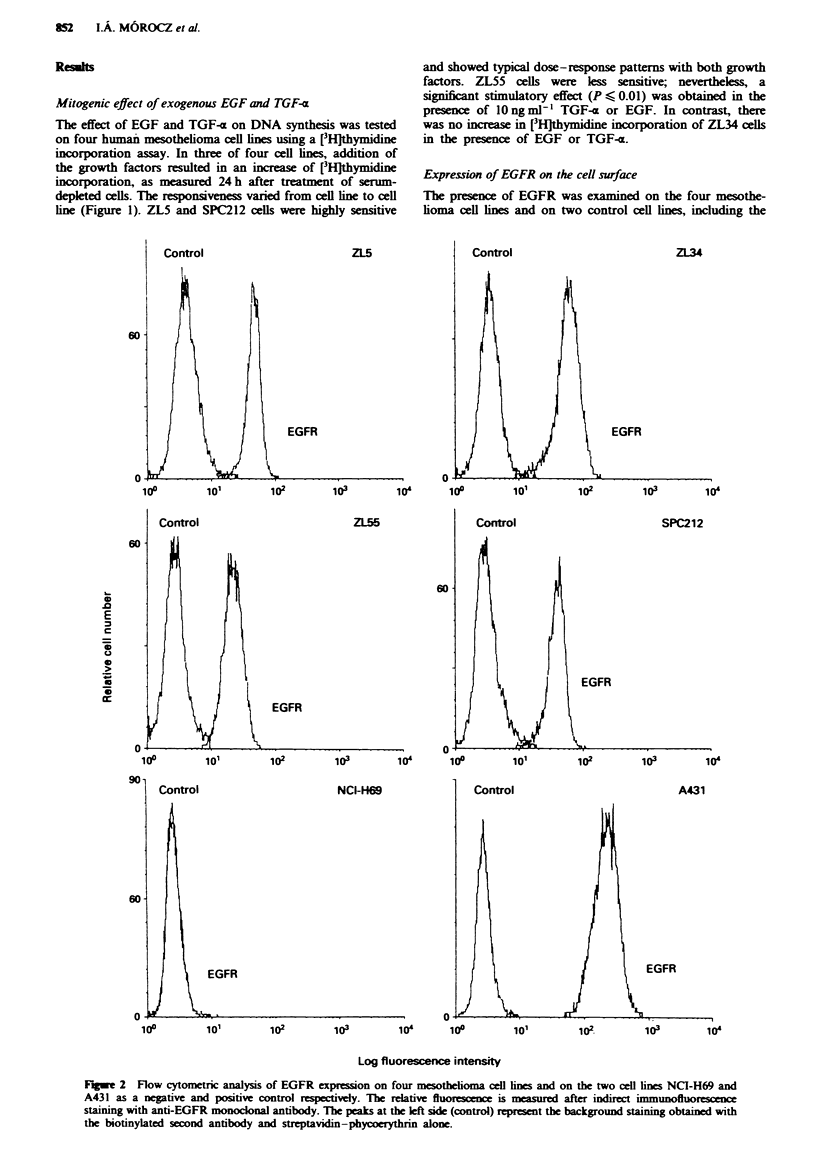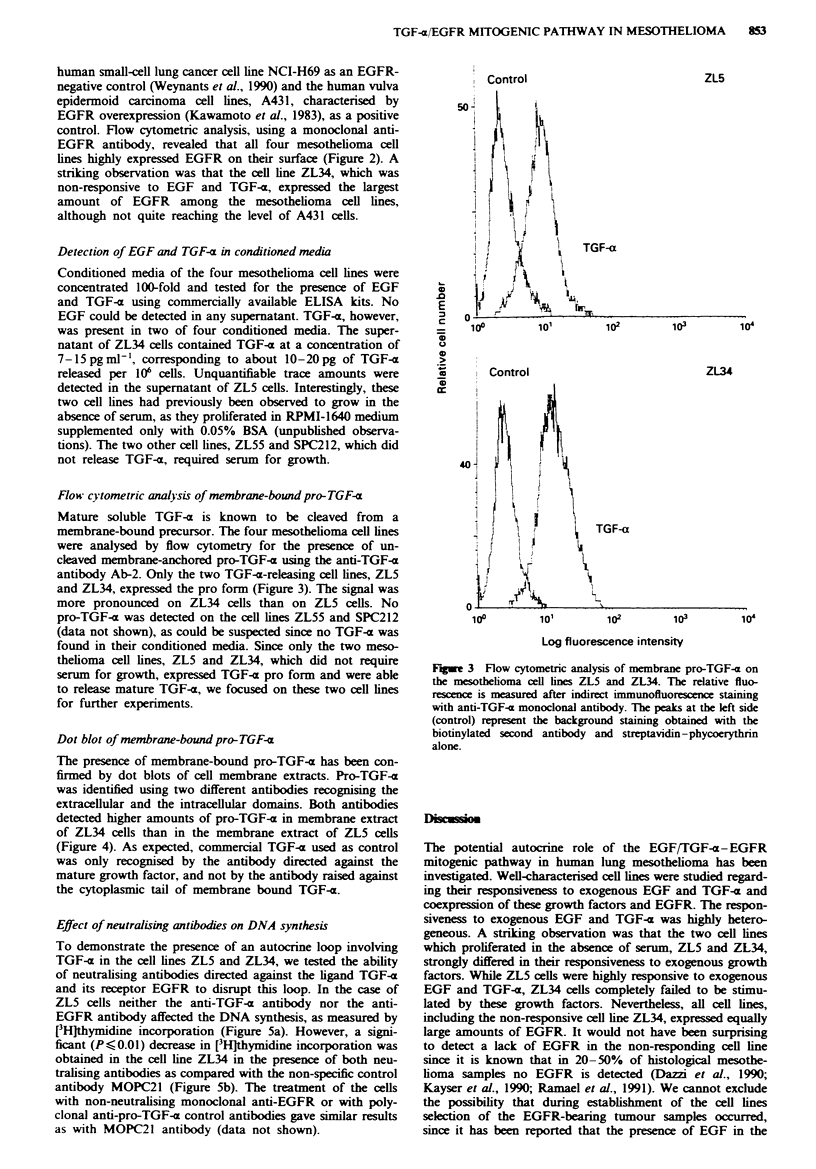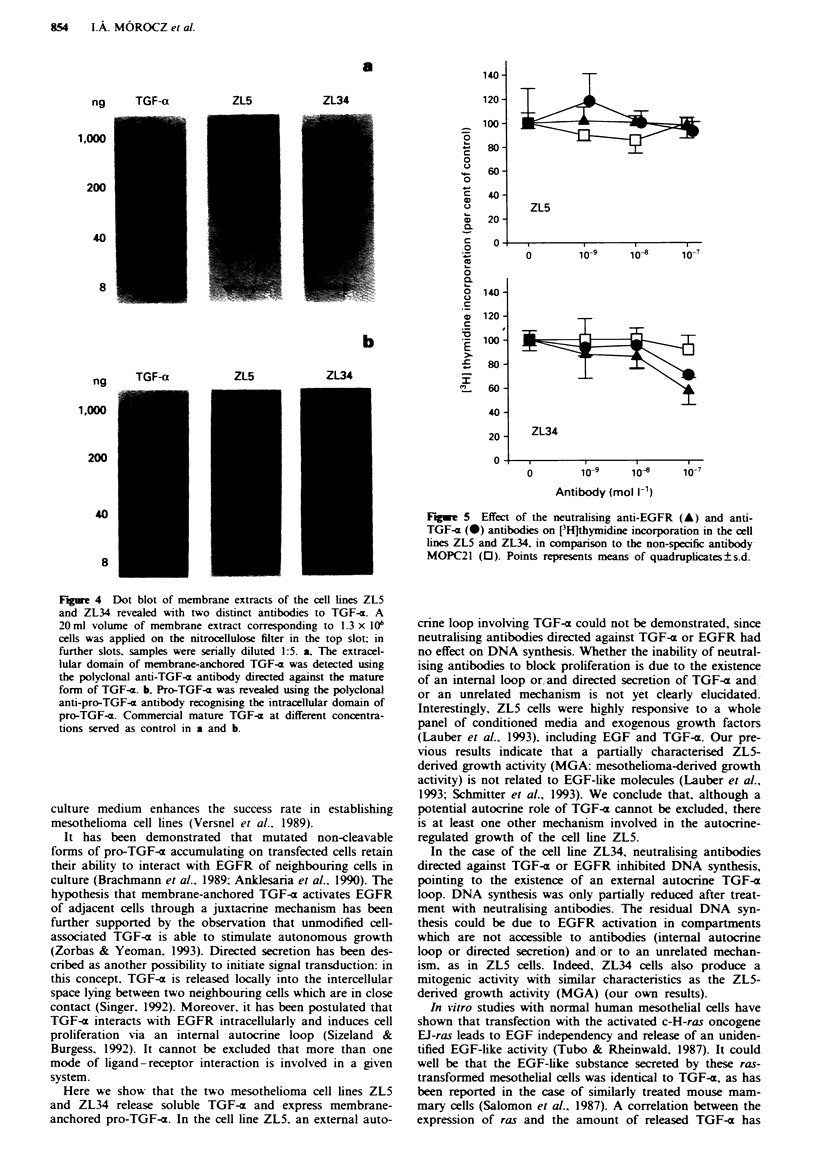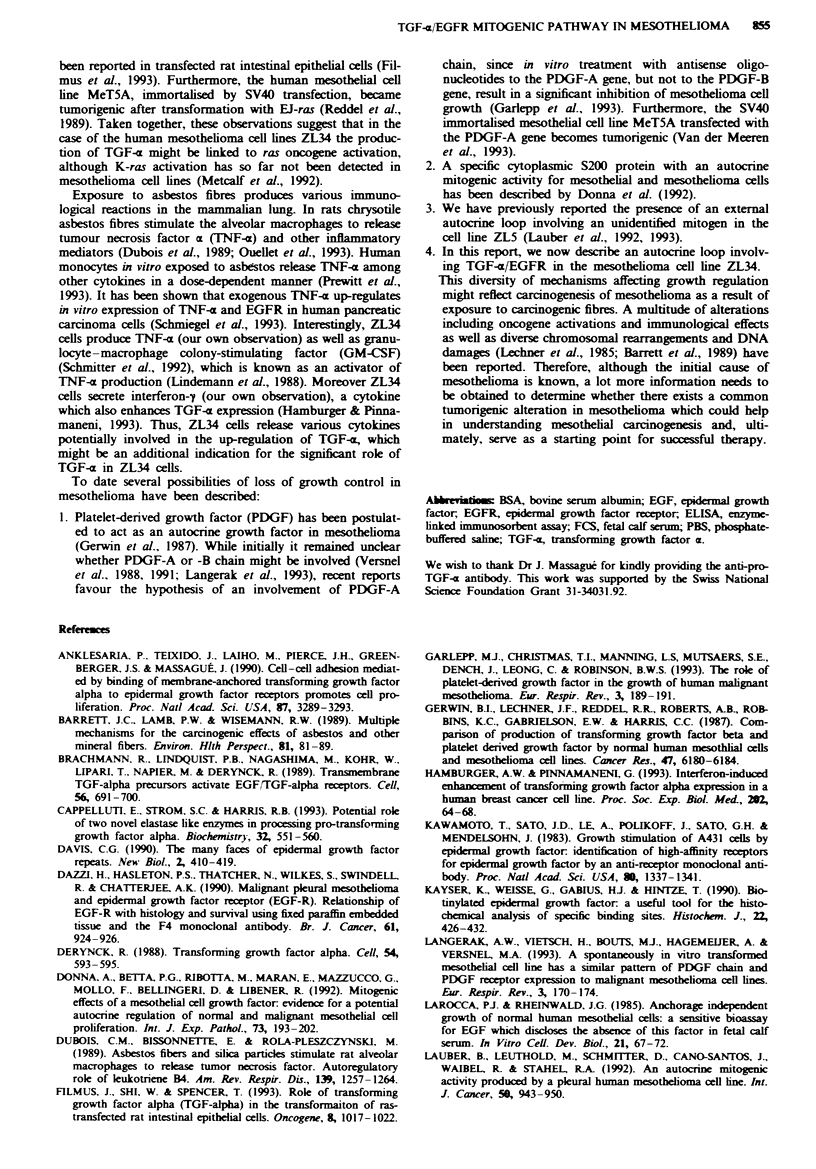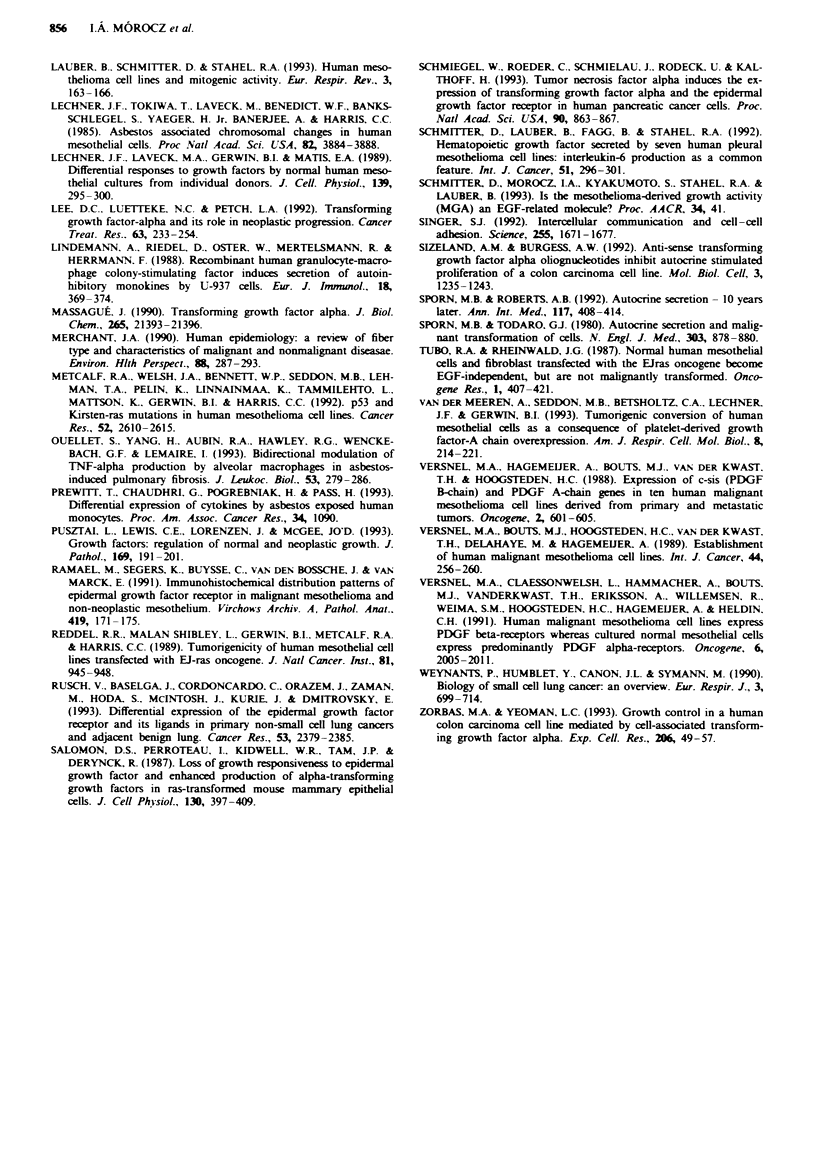# Autocrine stimulation of a human lung mesothelioma cell line is mediated through the transforming growth factor alpha/epidermal growth factor receptor mitogenic pathway.

**DOI:** 10.1038/bjc.1994.410

**Published:** 1994-11

**Authors:** I. A. Mórocz, D. Schmitter, B. Lauber, R. A. Stahel

**Affiliations:** Department of Medicine, University Hospital, Zurich, Switzerland.

## Abstract

**Images:**


Br. J. Cancer (1994), 70, 850-856 © Macmillan Press Ltd., 1994

### Autocrine stimulation of a human lung mesothelioma cell line is mediated through the transforming growth factor α/epidermal growth factor receptor mitogenic pathway

I.Á. Mórocz, D. Schmitter, B. Lauber & R.A. Stahel

*Division of Oncology, Department of Medicine, University Hospital CH-8091 Zurich, Switzerland.*

**Summary** Malignant cells frequently acquire a certain independency of exogenous growth factors via the coexpression of epidermal growth factor receptor (EGFR) and epidermal growth factor (EGF)-related molecules. In the present study we investigate a possible involvement of EGF-related molecules in the growth of human lung mesothelioma. Four well-characterised cell lines are analysed for their responsiveness to exogenous EGF and transforming growth factor α (TGF-α) as well as for coexpression of EGFR and EGF TGF-α. Both growth factors are able to stimulate DNA synthesis in three cell lines, although the degree of responsiveness is very variable, but neither EGF nor TGF-α has an effect on the cell line ZL34. In contrast, no heterogeneity is observed in the expression of EGFR, which is similarly high in all cell lines. Analysis of cell supernatants reveals that, whereas no EGF is detected, TGF-α is released by two cell lines. Furthermore, these two cell lines, ZL5 and ZL34, are shown to express the membrane anchored precursor pro-TGF-α. Thus, coexpression of EGFR and TGF-α is observed on two mesothelioma cell lines. The potential autocrine mitogenic role of TGF-α in these two cell lines was tested using neutralising antibodies against TGF-α and EGFR. In ZL5 cells DNA synthesis was not affected by the presence of neutralising antibodies, indicating that an external autocrine mitogenic pathway is not active in these cells. In ZL34 cells, however, the potential autocrine loop could be disrupted, as DNA synthesis was significantly reduced in the presence of neutralising antibodies. This result gives strong evidence for an autocrine role of TGF-α in the growth of the mesothelioma cell line ZL34.

Autonomous proliferation of malignant cells in culture is a frequently observed feature. This capacity may occur as a result of autocrine secretion of growth factors, circumventing the need for exogenous growth stimulatory signals. Proliferation in the absence of exogenous peptide growth factors, coexpression of growth factor and its receptor and inhibition of autonomous proliferation in the presence of specific neutralising antibody to the putative growth factor or its receptor are typical attributes of such cell lines. Since the statement of the autocrine hypothesis (Sporn & Todaro, 1980), the existence of these autocrine loops have been described for several different growth factors secreted by carcinoma derived cell lines (Sporn & Roberts, 1992; Pusztai *et al.*, 1993).

Mesothelioma, the tumour derived from mesothelial cells, is mainly associated with exposure to asbestos fibres, as well as to other natural or man-made carcinogenic fibres (Barrett *et al.*, 1989; Merchant, 1990). *In vitro* experiments using mesothelial cells and mesothelioma cell lines allow the study of growth conditions and mechanisms of these cells. For the culturing of human mesothelial cells, epidermal growth factor (EGF) is an essential element of the culture medium (La-Rocca & Rheinwald, 1985; Lechner *et al.*, 1989). EGF may facilitate the establishment of human mesothelioma cell lines, but it is not required for long-term culture (Versnel *et al.*, 1989). Among a large panel of defined growth factors tested, EGF has been described to exhibit a mitogenic activity on human mesothelioma cell lines (Lauber *et al.*, 1992, 1993).

The EGF family comprises a growing number of molecules related to EGF by amino acid sequence homology and or structural similarity (Davis, 1990). Transforming growth factor α (TGF-α ) is a member of this family (Derynck, 1988). The mature TGF-α molecule (50 amino acids) is released by proteolytic cleavage (Cappelluti *et al.*, 1993) from the precursor pro-TGF-α (160 amino acids), which is anchored in the cell membrane. There is increasing evidence that TGF-α plays an important role in the proliferation control of normal and neoplastic tissues (Lee *et al.*, 1992). TGF-α has been shown to bind to the EGF receptor (EGFR) in order to stimulate the proliferation of various carcinoma cell lines (Massagué, 1990). For autocrine activity coexpression of TGF-α and EGFR is required, a pattern observed in numerous tumour tissues and tumour-derived cell lines, e.g. co-expression of TGF-α and EGF was found in 38% of non-small-cell lung cancers (Rusch *et al.*, 1993).

We have shown in our previous studies that EGF has a mitogenic potential on mesothelioma cell lines (Lauber *et al.*, 1992, 1993). Several reports indicate that 50-80% of mesothelioma samples bear EGFR (Dazzi *et al.*, 1990; Kayser *et al.*, 1990; Ramael *et al.*, 1991). Considering these facts, we aimed to elucidate the possible autocrine role of the EGF/TGF-α -EGFR route in four human lung mesothelioma cell lines.

### Materials and methods

### *Cell cultures and media conditioning*

The pleural mesothelioma cell lines ZL5, ZL34, ZL55 and SPC212 were established from previously untreated patients as previously described (Schmitter *et al.*, 1992). Cells were routinely cultured in RPMI-1640 medium (Gibco Laboratories, Glasgow, UK) supplemented with 2 mM glutamine (Flow Laboratories, Irvine, UK) and 10% FCS (PAA, Linz, Austria) at 37°C in a humidified atmosphere with 5% carbon dioxide.

For production of conditioned media, subconfluent cells were washed twice with PBS and further cultured over 5 days in protein-free RPMI-1640 medium alone as described previously (Lauber *et al.*, 1992). Supernatants were spun at 700 *g* for 15 min and stored at −80°C.

### *[$^3$H]thymidine incorporation assays*

The mitogenic activity of growth factors and control media was determined by a [$^3$H]thymidine incorporation assay as described in detail elsewhere (Lauber *et al.*, 1992). Briefly, cells were grown in RPMI-1640/10% FCS medium for 2-3 days in 75 cm$^2$ culture flasks, washed twice with PBS and incubated in protein-free RPMI-1640 medium for 24 h. These

Correspondence: R.A. Stahel.
Received 25 January 1994; and in revised form 16 June 1994.

serum-depleted cells were then detached using trypsin 0.05% EDTA 0.02% (Seromed, Berlin, Germany) and further cultured in 96-well plates (2,000 cells per well) in RPMI-1640 medium alone for additional 24 h prior to the addition of test substances; 24 h after the addition of the test substances, [$^3$H]thymidine (1 μCi per well) was added as a 4 h pulse. Mitogenic activity was determined by comparison with the treatment of the cells with RPMI-1640 medium alone, defined as a mitogenic activity of 1.

Recombinant human EGF was purchased from Genzyme (Cambridge, MA, USA) and recombinant human TGF-α from Gibco (Gaithersburg, MD, USA).

### *Neutralisation assays*

Neutralisation assays were performed using two specific mouse monoclonal antibodies (Abs) of the IgG1 subclass: (i) the anti-human EGFR Ab (Genzyme no. 1209-00, Cambridge, MA, USA), recognising the extracellular domain of the EGFR and inhibiting the binding of the ligand to the receptor, and (ii) the anti-human TGF-α Ab (Ab-3, Oncogene Science no. GF15, Uniondale, NY, USA), interacting with residues 34–50 of TGF-α . Both antibodies exert their neutralising effect by spacial disruption of the pathway. The mouse monoclonal IgG1 antibody MOPC21 (Sigma no. M-9269) was used as a non-specific control. The mouse monoclonal antibody against EGFR (Pierce no. 36154X, Rockford, IL, USA) and the rabbit polyclonal anti-pro TGF-α Ab directed against the intracellular domain of pro-TGF-α (a gift from Dr Joan Massagué, Memorial Sloan-Kettering Cancer Center, New York, NY, USA) were used as non-neutralising control antibodies.

Serum-depleted cells were prepared as described above, and non-confluent cells (2,000 cells per well) were treated with antibodies in a concentration range from $10^{-7}$ to $10^{-9}$ M (1:100 to 1:2,500 dilution factor for the polyclonal antiserum). A 4 h pulse of [$^3$H]thymidine incorporation (1 μCi per well) was measured 24 h after treatment.

All experiments were performed in quadruplicates and repeated at least twice. Statistical analysis was performed by Student's *t*-test.

### *Flow cytometry*

For FACScan analysis cells were grown in RPMI-1640 10% FCS and detached by EDTA treatment (3 mM). A total of $5 \times 10^5$ cells were incubated on ice for 60–90 min in 500 μl of FACS buffer (PBS with 0.1% sodium azide and 1% BSA) containing one of the following mouse monoclonal antibodies at a concentration of $10^{-7}$ M: the anti-EGFR Ab (Genzyme) or the anti-human TGF-α Ab (Ab-2, Oncogene Science no. GF10); MOPC21 served as non-specific control antibody. After two cycles of washing (2 ml of FACS buffer) followed by centrifugation (250 *g*), biotinylated horse anti-mouse IgG Ab (Vector, Burlingame, CA, USA) was added for 30 min (500 μl diluted 1:200). Cells were washed and incubated with streptavidin–phycoerythrin (500 μl diluted 1:50; Becton-Dickinson, San José, CA, USA) for 30 min. After washing, cells were resuspended in 500 μl of FACS buffer and immediately analysed using a Becton Dickinson FACScan. Dead cells were excluded by propidiumiodide staining (1 μg ml$^{-1}$).

### *EGF and TGF-α quantification*

To detect EGF and TGF-α in conditioned media, commercial ELISA kits with a detection limit of 10 pg ml$^{-1}$ were used (EGF quantitative ELISA Assay no. QIA02 and TGF-α quantitative ELISA Assay no. QIA05, Oncogene Science, Uniondale, NY, USA). Conditioned media were concentrated by dialysis against 0.1 mM sodium chloride (dialysis membrane from Spectra Por, Houston, TX, USA; $M_r$ cut-off: 1,000 daltons) at 4°C over 5 days followed by Speed-vac evaporation. Dried samples were resuspended in sample buffer in order to obtain up to 100-fold concentrated conditioned media. The measurements were performed in duplicate with at least two batches of supernatants for each cell line.

### *Membrane extraction and immunoblotting*

Approximately $10^8$ cells grown in RPMI-1640 10% FCS and stored at −70°C were incubated for 40 min in 36 ml of lysis buffer (0.2 M HEPES, 1 mM EDTA, 0.1% BSA and 1 μM leupeptin from Sigma). After homogenisation in a manual glass homogeniser, a first centrifugation was performed at 1,000 *g* for 15 min in order to remove large cell particles. The supernatants were centrifuged for a second time at 50,000 *g* for 30 min. Pellets were resuspended in extraction buffer (lysis buffer containing 35 mM octyl-β-D-thioglucoside from Calbiochem, Behring Diagnostics, La Jolla, CA, USA) to a final volume of 1.5 ml and stored at −70°C. After thawing and an additional centrifugation at 10,000 *g* for 10 min, supernatant was applied by dotblotting on a nitrocellulose membrane (0.2 μm, Schleicher & Schuell, Keene, NH, USA), previously moistened with TBS (20 mM Tris–HCl, 1 M sodium chloride pH 7.5). Blotted membrane extract was fixed by a 30 min incubation in 1% glutaraldehyde in TBS. Non-specific binding was reduced by treating the membranes for 1 h with 5% non-fat milk (Bio-Rad Laboratories, Hercules, CA, USA) in TBS. Pro-TGF-α was detected using two antibodies recognising different domains: a polyclonal sheep anti-TGF-α Ab raised against mature human TGF-α (Biodesign, Kennebunkport, ME, USA) and a rabbit polyclonal anti-pro-TGF-α Ab directed against the intracellular domain of pro-TGF-α (a gift from Dr Joan Massagué). Antibodies were diluted (1:1,000 for the sheep antibody and 1:5,000 for the rabbit antibody) in antibody buffer (5% non-fat milk, 1% gelatine, 0.5% Tween-20 in TBS). Membranes were incubated in the antibody solution for 1 h, and thereafter washed three times with antibody buffer. Affinity-purified donkey anti-sheep antibody (diluted 1:5,000) or goat anti-rabbit F(ab')$_2$ fragment (diluted 1:3,000), both conjugated to alkaline phosphatase (Jackson ImmunoResearch, Milan Analytica, La Roche, Switzerland), were used as second antibodies. After three wash steps with antibody buffer followed by two wash steps with substrate buffer (4 mM magnesium chloride, 0.1 M diethyl barbituric acid sodium salt, from Fluka, Buchs, Switzerland), the substrate cocktail [BCIP NBT alkaline phosphatase colour development solution, following the manufacturer's (BioRad) instructions] was added for 45 min at 37°C.

**Figure 1** Dose–response patterns of the cell lines ZL5, ZL34, ZL55 and SPC212 to EGF (○) and TGF-α (●). The mitogenic activity is defined as [$^3$H]thymidine incorporation 24 h after treatment of the cells with growth factor as compared with untreated control. Points represent means of quadruplicates ± s.d.

### Results

### *Mitogenic effect of exogenous EGF and TGF-α*

The effect of EGF and TGF-α on DNA synthesis was tested on four human mesothelioma cell lines using a [$^3$H]thymidine incorporation assay. In three of four cell lines, addition of the growth factors resulted in an increase of [$^3$H]thymidine incorporation, as measured 24 h after treatment of serum-depleted cells. The responsiveness varied from cell line to cell line (Figure 1). ZL5 and SPC212 cells were highly sensitive and showed typical dose–response patterns with both growth factors. ZL55 cells were less sensitive; nevertheless, a significant stimulatory effect ($P \leqslant 0.01$) was obtained in the presence of 10 ng ml$^{-1}$ TGF-α or EGF. In contrast, there was no increase in [$^3$H]thymidine incorporation of ZL34 cells in the presence of EGF or TGF-α.

### *Expression of EGFR on the cell surface*

The presence of EGFR was examined on the four mesothelioma cell lines and on two control cell lines, including the

**Figure 2** Flow cytometric analysis of EGFR expression on four mesothelioma cell lines and on the two cell lines NCI-H69 and A431 as a negative and positive control respectively. The relative fluorescence is measured after indirect immunofluorescence staining with anti-EGFR monoclonal antibody. The peaks at the left side (control) represent the background staining obtained with the biotinylated second antibody and streptavidin–phycoerythrin alone.

human small-cell lung cancer cell line NCI-H69 as an EGFR-negative control (Weynants *et al.*, 1990) and the human vulva epidermoid carcinoma cell lines, A431, characterised by EGFR overexpression (Kawamoto *et al.*, 1983), as a positive control. Flow cytometric analysis, using a monoclonal anti-EGFR antibody, revealed that all four mesothelioma cell lines highly expressed EGFR on their surface (Figure 2). A striking observation was that the cell line ZL34, which was non-responsive to EGF and TGF-α, expressed the largest amount of EGFR among the mesothelioma cell lines, although not quite reaching the level of A431 cells.

*Detection of EGF and TGF-α in conditioned media*

Conditioned media of the four mesothelioma cell lines were concentrated 100-fold and tested for the presence of EGF and TGF-α using commercially available ELISA kits. No EGF could be detected in any supernatant. TGF-α, however, was present in two of four conditioned media. The supernatant of ZL34 cells contained TGF-α at a concentration of 7–15 pg $ml^{-1}$, corresponding to about 10–20 pg of TGF-α released per $10^6$ cells. Unquantifiable trace amounts were detected in the supernatant of ZL5 cells. Interestingly, these two cell lines had previously been observed to grow in the absence of serum, as they proliferated in RPMI-1640 medium supplemented only with 0.05% BSA (unpublished observations). The two other cell lines, ZL55 and SPC212, which did not release TGF-α, required serum for growth.

*Flow cytometric analysis of membrane-bound pro-TGF-α*

Mature soluble TGF-α is known to be cleaved from a membrane-bound precursor. The four mesothelioma cell lines were analysed by flow cytometry for the presence of uncleaved membrane-anchored pro-TGF-α using the anti-TGF-α antibody Ab-2. Only the two TGF-α-releasing cell lines, ZL5 and ZL34, expressed the pro form (Figure 3). The signal was more pronounced on ZL34 cells than on ZL5 cells. No pro-TGF-α was detected on the cell lines ZL55 and SPC212 (data not shown), as could be suspected since no TGF-α was found in their conditioned media. Since only the two mesothelioma cell lines, ZL5 and ZL34, which did not require serum for growth, expressed TGF-α pro form and were able to release mature TGF-α, we focused on these two cell lines for further experiments.

*Dot blot of membrane-bound pro-TGF-α*

The presence of membrane-bound pro-TGF-α has been confirmed by dot blots of cell membrane extracts. Pro-TGF-α was identified using two different antibodies recognising the extracellular and the intracellular domains. Both antibodies detected higher amounts of pro-TGF-α in membrane extract of ZL34 cells than in the membrane extract of ZL5 cells (Figure 4). As expected, commercial TGF-α used as control was only recognised by the antibody directed against the mature growth factor, and not by the antibody raised against the cytoplasmic tail of membrane bound TGF-α.

*Effect of neutralising antibodies on DNA synthesis*

To demonstrate the presence of an autocrine loop involving TGF-α in the cell lines ZL5 and ZL34, we tested the ability of neutralising antibodies directed against the ligand TGF-α and its receptor EGFR to disrupt this loop. In the case of ZL5 cells neither the anti-TGF-α antibody nor the anti-EGFR antibody affected the DNA synthesis, as measured by [$^3$H]thymidine incorporation (Figure 5a). However, a significant ($P \leqslant 0.01$) decrease in [$^3$H]thymidine incorporation was obtained in the cell line ZL34 in the presence of both neutralising antibodies as compared with the non-specific control antibody MOPC21 (Figure 5b). The treatment of the cells with non-neutralising monoclonal anti-EGFR or with polyclonal anti-pro-TGF-α control antibodies gave similar results as with MOPC21 antibody (data not shown).

**Figure 3** Flow cytometric analysis of membrane pro-TGF-α on the mesothelioma cell lines ZL5 and ZL34. The relative fluorescence is measured after indirect immunofluorescence staining with anti-TGF-α monoclonal antibody. The peaks at the left side (control) represent the background staining obtained with the biotinylated second antibody and streptavidin-phycoerythrin alone.

### Discussion

The potential autocrine role of the EGF/TGF-α–EGFR mitogenic pathway in human lung mesothelioma has been investigated. Well-characterised cell lines were studied regarding their responsiveness to exogenous EGF and TGF-α and coexpression of these growth factors and EGFR. The responsiveness to exogenous EGF and TGF-α was highly heterogeneous. A striking observation was that the two cell lines which proliferated in the absence of serum, ZL5 and ZL34, strongly differed in their responsiveness to exogenous growth factors. While ZL5 cells were highly responsive to exogenous EGF and TGF-α, ZL34 cells completely failed to be stimulated by these growth factors. Nevertheless, all cell lines, including the non-responsive cell line ZL34, expressed equally large amounts of EGFR. It would not have been surprising to detect a lack of EGFR in the non-responding cell line since it is known that in 20–50% of histological mesothelioma samples no EGFR is detected (Dazzi *et al.*, 1990; Kayser *et al.*, 1990; Ramael *et al.*, 1991). We cannot exclude the possibility that during establishment of the cell lines selection of the EGFR-bearing tumour samples occurred, since it has been reported that the presence of EGF in the

**Figure 4** Dot blot of membrane extracts of the cell lines ZL5 and ZL34 revealed with two distinct antibodies to TGF-α. A 20 ml volume of membrane extract corresponding to 1.3 × $10^6$ cells was applied on the nitrocellulose filter in the top slot; in further slots, samples were serially diluted 1:5. **a**, The extracellular domain of membrane-anchored TGF-α was detected using the polyclonal anti-TGF-α antibody directed against the mature form of TGF-α. **b**, Pro-TGF-α was revealed using the polyclonal anti-pro-TGF-α antibody recognising the intracellular domain of pro-TGF-α. Commercial mature TGF-α at different concentrations served as control in **a** and **b**.

**Figure 5** Effect of the neutralising anti-EGFR (▲) and anti-TGF-α (●) antibodies on [$^3$H]thymidine incorporation in the cell lines ZL5 and ZL34, in comparison to the non-specific antibody MOPC21 (□). Points represents means of quadruplicates ± s.d.

culture medium enhances the success rate in establishing mesothelioma cell lines (Versnel *et al*., 1989).

It has been demonstrated that mutated non-cleavable forms of pro-TGF-α accumulating on transfected cells retain their ability to interact with EGFR of neighbouring cells in culture (Brachmann *et al*., 1989; Anklesaria *et al*., 1990). The hypothesis that membrane-anchored TGF-α activates EGFR of adjacent cells through a juxtacrine mechanism has been further supported by the observation that unmodified cell-associated TGF-α is able to stimulate autonomous growth (Zorbas & Yeoman, 1993). Directed secretion has been described as another possibility to initiate signal transduction: in this concept, TGF-α is released locally into the intercellular space lying between two neighbouring cells which are in close contact (Singer, 1992). Moreover, it has been postulated that TGF-α interacts with EGFR intracellularly and induces cell proliferation via an internal autocrine loop (Sizeland & Burgess, 1992). It cannot be excluded that more than one mode of ligand–receptor interaction is involved in a given system.

Here we show that the two mesothelioma cell lines ZL5 and ZL34 release soluble TGF-α and express membrane-anchored pro-TGF-α. In the cell line ZL5, an external autocrine loop involving TGF-α could not be demonstrated, since neutralising antibodies directed against TGF-α or EGFR had no effect on DNA synthesis. Whether the inability of neutralising antibodies to block proliferation is due to the existence of an internal loop or/and directed secretion of TGF-α and/or an unrelated mechanism is not yet clearly elucidated. Interestingly, ZL5 cells were highly responsive to a whole panel of conditioned media and exogenous growth factors (Lauber *et al*., 1993), including EGF and TGF-α. Our previous results indicate that a partially characterised ZL5-derived growth activity (MGA: mesothelioma-derived growth activity) is not related to EGF-like molecules (Lauber *et al*., 1993; Schmitter *et al*., 1993). We conclude that, although a potential autocrine role of TGF-α cannot be excluded, there is at least one other mechanism involved in the autocrine-regulated growth of the cell line ZL5.

In the case of the cell line ZL34, neutralising antibodies directed against TGF-α or EGFR inhibited DNA synthesis, pointing to the existence of an external autocrine TGF-α loop. DNA synthesis was only partially reduced after treatment with neutralising antibodies. The residual DNA synthesis could be due to EGFR activation in compartments which are not accessible to antibodies (internal autocrine loop or directed secretion) and/or to an unrelated mechanism, as in ZL5 cells. Indeed, ZL34 cells also produce a mitogenic activity with similar characteristics as the ZL5-derived growth activity (MGA) (our own results).

*In vitro* studies with normal human mesothelial cells have shown that transfection with the activated c-H-*ras* oncogene EJ-*ras* leads to EGF independency and release of an unidentified EGF-like activity (Tubo & Rheinwald, 1987). It could well be that the EGF-like substance secreted by these *ras*-transformed mesothelial cells was identical to TGF-α, as has been reported in the case of similarly treated mouse mammary cells (Salomon *et al*., 1987). A correlation between the expression of *ras* and the amount of released TGF-α has been reported in transfected rat intestinal epithelial cells (Filmus *et al.*, 1993). Furthermore, the human mesothelial cell line MeT5A, immortalised by SV40 transfection, became tumorigenic after transformation with EJ-*ras* (Reddel *et al.*, 1989). Taken together, these observations suggest that in the case of the human mesothelioma cell lines ZL34 the production of TGF-α might be linked to *ras* oncogene activation, although K-*ras* activation has so far not been detected in mesothelioma cell lines (Metcalf *et al.*, 1992).

Exposure to asbestos fibres produces various immunological reactions in the mammalian lung. In rats chrysotile asbestos fibres stimulate the alveolar macrophages to release tumour necrosis factor α (TNF-α) and other inflammatory mediators (Dubois *et al.*, 1989; Ouellet *et al.*, 1993). Human monocytes *in vitro* exposed to asbestos release TNF-α among other cytokines in a dose-dependent manner (Prewitt *et al.*, 1993). It has been shown that exogenous TNF-α up-regulates *in vitro* expression of TNF-α and EGFR in human pancreatic carcinoma cells (Schmiegel *et al.*, 1993). Interestingly, ZL34 cells produce TNF-α (our own observation) as well as granulocyte–macrophage colony-stimulating factor (GM-CSF) (Schmitter *et al.*, 1992), which is known as an activator of TNF-α production (Lindemann *et al.*, 1988). Moreover ZL34 cells secrete interferon-γ (our own observation), a cytokine which also enhances TGF-α expression (Hamburger & Pinnamaneni, 1993). Thus, ZL34 cells release various cytokines potentially involved in the up-regulation of TGF-α, which might be an additional indication for the significant role of TGF-α in ZL34 cells.

To date several possibilities of loss of growth control in mesothelioma have been described:

1. Platelet-derived growth factor (PDGF) has been postulated to act as an autocrine growth factor in mesothelioma (Gerwin *et al.*, 1987). While initially it remained unclear whether PDGF-A or -B chain might be involved (Versnel *et al.*, 1988, 1991; Langerak *et al.*, 1993), recent reports favour the hypothesis of an involvement of PDGF-A chain, since *in vitro* treatment with antisense oligonucleotides to the PDGF-A gene, but not to the PDGF-B gene, result in a significant inhibition of mesothelioma cell growth (Garlepp *et al.*, 1993). Furthermore, the SV40 immortalised mesothelial cell line MeT5A transfected with the PDGF-A gene becomes tumorigenic (Van der Meeren *et al.*, 1993).
2. A specific cytoplasmic S200 protein with an autocrine mitogenic activity for mesothelial and mesothelioma cells has been described by Donna *et al.* (1992).
3. We have previously reported the presence of an external autocrine loop involving an unidentified mitogen in the cell line ZL5 (Lauber *et al.*, 1992, 1993).
4. In this report, we now describe an autocrine loop involving TGF-α/EGFR in the mesothelioma cell line ZL34.

This diversity of mechanisms affecting growth regulation might reflect carcinogenesis of mesothelioma as a result of exposure to carcinogenic fibres. A multitude of alterations including oncogene activations and immunological effects as well as diverse chromosomal rearrangements and DNA damages (Lechner *et al.*, 1985; Barrett *et al.*, 1989) have been reported. Therefore, although the initial cause of mesothelioma is known, a lot more information needs to be obtained to determine whether there exists a common tumorigenic alteration in mesothelioma which could help in understanding mesothelial carcinogenesis and, ultimately, serve as a starting point for successful therapy.

**Abbreviations:** BSA, bovine serum albumin; EGF, epidermal growth factor; EGFR, epidermal growth factor receptor; ELISA, enzyme-linked immunosorbent assay; FCS, fetal calf serum; PBS, phosphate-buffered saline; TGF-α, transforming growth factor α.

We wish to thank Dr J. Massagué for kindly providing the anti-pro-TGF-α antibody. This work was supported by the Swiss National Science Foundation Grant 31-34031.92.

### References

ANKLESARIA, P., TEIXIDO, J., LAIHO, M., PIERCE, J.H., GREENBERGER, J.S. & MASSAGUÉ, J. (1990). Cell–cell adhesion mediated by binding of membrane-anchored transforming growth factor alpha to epidermal growth factor receptors promotes cell proliferation. *Proc. Natl Acad. Sci. USA*, **87**, 3289–3293.

BARRETT, J.C., LAMB, P.W. & WISEMANN, R.W. (1989). Multiple mechanisms for the carcinogenic effects of asbestos and other mineral fibers. *Environ. Hlth Perspect.*, **81**, 81–89.

BRACHMANN, R., LINDQUIST, P.B., NAGASHIMA, M., KOHR, W., LIPARI, T., NAPIER, M. & DERYNCK, R. (1989). Transmembrane TGF-alpha precursors activate EGF/TGF-alpha receptors. *Cell*, **56**, 691–700.

CAPPELLUTI, E., STROM, S.C. & HARRIS, R.B. (1993). Potential role of two novel elastase like enzymes in processing pro-transforming growth factor alpha. *Biochemistry*, **32**, 551–560.

DAVIS, C.G. (1990). The many faces of epidermal growth factor repeats. *New Biol.*, **2**, 410–419.

DAZZI, H., HASLETON, P.S., THATCHER, N., WILKES, S., SWINDELL, R. & CHATTERJEE, A.K. (1990). Malignant pleural mesothelioma and epidermal growth factor receptor (EGF-R). Relationship of EGF-R with histology and survival using fixed paraffin embedded tissue and the F4 monoclonal antibody. *Br. J. Cancer*, **61**, 924–926.

DERYNCK, R. (1988). Transforming growth factor alpha. *Cell*, **54**, 593–595.

DONNA, A., BETTA, P.G., RIBOTTA, M., MARAN, E., MAZZUCCO, G., MOLLO, F., BELLINGERI, D. & LIBENER, R. (1992). Mitogenic effects of a mesothelial cell growth factor: evidence for a potential autocrine regulation of normal and malignant mesothelial cell proliferation. *Int. J. Exp. Pathol.*, **73**, 193–202.

DUBOIS, C.M., BISSONNETTE, E. & ROLA-PLESZCZYNSKI, M. (1989). Asbestos fibers and silica particles stimulate rat alveolar macrophages to release tumor necrosis factor. Autoregulatory role of leukotriene B4. *Am. Rev. Respir. Dis.*, **139**, 1257–1264.

FILMUS, J., SHI, W. & SPENCER, T. (1993). Role of transforming growth factor alpha (TGF-alpha) in the transformaiton of ras-transfected rat intestinal epithelial cells. *Oncogene*, **8**, 1017–1022.

GARLEPP, M.J., CHRISTMAS, T.I., MANNING, L.S, MUTSAERS, S.E., DENCH, J., LEONG, C. & ROBINSON, B.W.S. (1993). The role of platelet-derived growth factor in the growth of human malignant mesothelioma. *Eur. Respir. Rev.*, **3**, 189–191.

GERWIN, B.I., LECHNER, J.F., REDDEL, R.R., ROBERTS, A.B., ROBBINS, K.C., GABRIELSON, E.W. & HARRIS, C.C. (1987). Comparison of production of transforming growth factor beta and platelet derived growth factor by normal human mesothlial cells and mesothelioma cell lines. *Cancer Res.*, **47**, 6180–6184.

HAMBURGER, A.W. & PINNAMANENI, G. (1993). Interferon-induced enhancement of transforming growth factor alpha expression in a human breast cancer cell line. *Proc. Soc. Exp. Biol. Med.*, **202**, 64–68.

KAWAMOTO, T., SATO, J.D., LE, A., POLIKOFF, J., SATO, G.H. & MENDELSOHN, J. (1983). Growth stimulation of A431 cells by epidermal growth factor: identification of high-affinity receptors for epidermal growth factor by an anti-receptor monoclonal antibody. *Proc. Natl Acad. Sci. USA*, **80**, 1337–1341.

KAYSER, K., WEISSE, G., GABIUS, H.J. & HINTZE, T. (1990). Biotinylated epidermal growth factor: a useful tool for the histochemical analysis of specific binding sites. *Histochem. J.*, **22**, 426–432.

LANGERAK, A.W., VIETSCH, H., BOUTS, M.J., HAGEMEIJER, A. & VERSNEL, M.A. (1993). A spontaneously in vitro transformed mesothelial cell line has a similar pattern of PDGF chain and PDGF receptor expression to malignant mesothelioma cell lines. *Eur. Respir. Rev.*, **3**, 170–174.

LAROCCA, P.J. & RHEINWALD, J.G. (1985). Anchorage independent growth of normal human mesothelial cells: a sensitive bioassay for EGF which discloses the absence of this factor in fetal calf serum. *In Vitro Cell. Dev. Biol.*, **21**, 67–72.

LAUBER, B., LEUTHOLD, M., SCHMITTER, D., CANO-SANTOS, J., WAIBEL, R. & STAHEL, R.A. (1992). An autocrine mitogenic activity produced by a pleural human mesothelioma cell line. *Int. J. Cancer*, **50**, 943–950.

LAUBER, B., SCHMITTER, D. & STAHEL, R.A. (1993). Human mesothelioma cell lines and mitogenic activity. *Eur. Respir. Rev.*, **3**, 163-166.

LECHNER, J.F., TOKIWA, T., LAVECK, M., BENEDICT, W.F., BANKS-SCHLEGEL, S., YAEGER, H. Jr, BANERJEE, A. & HARRIS, C.C. (1985). Asbestos associated chromosomal changes in human mesothelial cells. *Proc Natl Acad. Sci. USA*, **82**, 3884-3888.

LECHNER, J.F., LAVECK, M.A., GERWIN, B.I. & MATIS, E.A. (1989). Differential responses to growth factors by normal human mesothelial cultures from individual donors. *J. Cell. Physiol.*, **139**, 295-300.

LEE, D.C., LUETTEKE, N.C. & PETCH, L.A. (1992). Transforming growth factor-alpha and its role in neoplastic progression. *Cancer Treat. Res.*, **63**, 233-254.

LINDEMANN, A., RIEDEL, D., OSTER, W., MERTELSMANN, R. & HERRMANN, F. (1988). Recombinant human granulocyte-macrophage colony-stimulating factor induces secretion of autoinhibitory monokines by U-937 cells. *Eur. J. Immunol.*, **18**, 369-374.

MASSAGUÉ, J. (1990). Transforming growth factor alpha. *J. Biol. Chem.*, **265**, 21393-21396.

MERCHANT, J.A. (1990). Human epidemiology: a review of fiber type and characteristics of malignant and nonmalignant disease. *Environ. Hlth Perspect.*, **88**, 287-293.

METCALF, R.A., WELSH, J.A., BENNETT, W.P., SEDDON, M.B., LEHMAN, T.A., PELIN, K., LINNAINMAA, K., TAMMILEHTO, L., MATTSON, K., GERWIN, B.I. & HARRIS, C.C. (1992). p53 and Kirsten-ras mutations in human mesothelioma cell lines. *Cancer Res.*, **52**, 2610-2615.

OUELLET, S., YANG, H., AUBIN, R.A., HAWLEY, R.G., WENCKEBACH, G.F. & LEMAIRE, I. (1993). Bidirectional modulation of TNF-alpha production by alveolar macrophages in asbestos-induced pulmonary fibrosis. *J. Leukoc. Biol.*, **53**, 279-286.

PREWITT, T., CHAUDHRI, G., POGREBNIAK, H. & PASS, H. (1993). Differential expression of cytokines by asbestos exposed human monocytes. *Proc. Am. Assoc. Cancer Res.*, **34**, 1090.

PUSZTAI, L., LEWIS, C.E., LORENZEN, J. & MCGEE, JO'D. (1993). Growth factors: regulation of normal and neoplastic growth. *J. Pathol.*, **169**, 191-201.

RAMAEL, M., SEGERS, K., BUYSSE, C., VAN DEN BOSSCHE, J. & VAN MARCK, E. (1991). Immunohistochemical distribution patterns of epidermal growth factor receptor in malignant mesothelioma and non-neoplastic mesothelium. *Virchows Archiv. A, Pathol. Anat.*, **419**, 171-175.

REDDEL, R.R., MALAN SHIBLEY, L., GERWIN, B.I., METCALF, R.A. & HARRIS, C.C. (1989). Tumorigenicity of human mesothelial cell lines transfected with EJ-ras oncogene. *J. Natl Cancer. Inst.*, **81**, 945-948.

RUSCH, V., BASELGA, J., CORDONCARDO, C., ORAZEM, J., ZAMAN, M., HODA, S., MCINTOSH, J., KURIE, J. & DMITROVSKY, E. (1993). Differential expression of the epidermal growth factor receptor and its ligands in primary non-small cell lung cancers and adjacent benign lung. *Cancer Res.*, **53**, 2379-2385.

SALOMON, D.S., PERROTEAU, I., KIDWELL, W.R., TAM, J.P. & DERYNCK, R. (1987). Loss of growth responsiveness to epidermal growth factor and enhanced production of alpha-transforming growth factors in ras-transformed mouse mammary epithelial cells. *J. Cell Physiol.*, **130**, 397-409.

SCHMIEGEL, W., ROEDER, C., SCHMIELAU, J., RODECK, U. & KALTHOFF, H. (1993). Tumor necrosis factor alpha induces the expression of transforming growth factor alpha and the epidermal growth factor receptor in human pancreatic cancer cells. *Proc. Natl Acad. Sci. USA*, **90**, 863-867.

SCHMITTER, D., LAUBER, B., FAGG, B. & STAHEL, R.A. (1992). Hematopoietic growth factor secreted by seven human pleural mesothelioma cell lines: interleukin-6 production as a common feature. *Int. J. Cancer*, **51**, 296-301.

SCHMITTER, D., MOROCZ, I.A., KYAKUMOTO, S., STAHEL, R.A. & LAUBER, B. (1993). Is the mesothelioma-derived growth activity (MGA) an EGF-related molecule? *Proc. AACR*, **34**, 41.

SINGER, S.J. (1992). Intercellular communication and cell-cell adhesion. *Science*, **255**, 1671-1677.

SIZELAND, A.M. & BURGESS, A.W. (1992). Anti-sense transforming growth factor alpha oliognucleotides inhibit autocrine stimulated proliferation of a colon carcinoma cell line. *Mol. Biol. Cell*, **3**, 1235-1243.

SPORN, M.B. & ROBERTS, A.B. (1992). Autocrine secretion - 10 years later. *Ann. Int. Med.*, **117**, 408-414.

SPORN, M.B. & TODARO, G.J. (1980). Autocrine secretion and malignant transformation of cells. *N. Engl. J. Med.*, **303**, 878-880.

TUBO, R.A. & RHEINWALD, J.G. (1987). Normal human mesothelial cells and fibroblast transfected with the EJras oncogene become EGF-independent, but are not malignantly transformed. *Oncogene Res.*, **1**, 407-421.

VAN DER MEEREN, A., SEDDON, M.B., BETSHOLTZ, C.A., LECHNER, J.F. & GERWIN, B.I. (1993). Tumorigenic conversion of human mesothelial cells as a consequence of platelet-derived growth factor-A chain overexpression. *Am. J. Respir. Cell. Mol. Biol.*, **8**, 214-221.

VERSNEL, M.A., HAGEMEIJER, A., BOUTS, M.J., VAN DER KWAST, T.H. & HOOGSTEDEN, H.C. (1988). Expression of c-sis (PDGF B-chain) and PDGF A-chain genes in ten human malignant mesothelioma cell lines derived from primary and metastatic tumors. *Oncogene*, **2**, 601-605.

VERSNEL, M.A., BOUTS, M.J., HOOGSTEDEN, H.C., VAN DER KWAST, T.H., DELAHAYE, M. & HAGEMEIJER, A. (1989). Establishment of human malignant mesothelioma cell lines. *Int. J. Cancer*, **44**, 256-260.

VERSNEL, M.A., CLAESSONWELSH, L., HAMMACHER, A., BOUTS, M.J., VANDERKWAST, T.H., ERIKSSON, A., WILLEMSEN, R., WEIMA, S.M., HOOGSTEDEN, H.C., HAGEMEIJER, A. & HELDIN, C.H. (1991). Human malignant mesothelioma cell lines express PDGF beta-receptors whereas cultured normal mesothelial cells express predominantly PDGF alpha-receptors. *Oncogene*, **6**, 2005-2011.

WEYNANTS, P., HUMBLET, Y., CANON, J.L. & SYMANN, M. (1990). Biology of small cell lung cancer: an overview. *Eur. Respir. J.*, **3**, 699-714.

ZORBAS, M.A. & YEOMAN, L.C. (1993). Growth control in a human colon carcinoma cell line mediated by cell-associated transforming growth factor alpha. *Exp. Cell. Res.*, **206**, 49-57.